# The effects of yoga and quiet rest on subjective levels of anxiety and physiological correlates: a 2-way crossover randomized trial

**DOI:** 10.1186/s12906-018-2343-1

**Published:** 2018-10-17

**Authors:** Kembra Albracht-Schulte, Jacalyn Robert-McComb

**Affiliations:** 1 Department of Nutritional Sciences, Texas Tech University, Lubbock, TX USA; 2 Department of Kinesiology and Sport Management, Texas Tech University, 3204 Main Street, Box 43011, Lubbock, TX 79407-3011 USA

**Keywords:** Affect, Autonomic function, Emotion, Heart rate variability, Quiet rest

## Abstract

**Background:**

Rest or acute exercise can decrease state anxiety, with some evidence showing exercise to prevent laboratory-induced elevations in anxiety. No study has examined whether yoga provides short-term protection against laboratory-induced anxiety. The aim of this study was to examine the effectiveness of an acute YogaFit session on state anxiety and measures of heart rate variability (HRV) to determine whether yoga provides short-term protection against emotional picture stimuli.

**Methods:**

A randomized repeated-measures crossover clinical trial was performed. Forty healthy, female college students completed a 30 min session of YogaFit and a time-matched seated rest condition on separate days. After each condition, participants viewed 30 min of emotional picture stimuli. State anxiety, heart rate and time-domain and frequency-domain measures of HRV were assessed baseline, post- condition, and post-exposure to emotional stimuli. Data were analysed using a condition x time (2 × 3) repeated-measures ANOVA.

**Results:**

Post-hoc comparisons indicate the following: (1) state anxiety significantly decreased from baseline to post-condition for both yoga and rest (*p* = 0.001) but returned to baseline values following exposure to emotional stimuli (*p* < 0.001) for both conditions; (2) heart rate decreased post-condition to post-exposure (*p* = 0.020) and baseline to post-exposure (*p* = 0.033) for both conditions; (3) time-domain measure of HRV showed a significant increase in HRV between baseline and post-condition (*p* = 0 .019), post-condition and post-exposure (*p* = 0 .007), and between baseline and post-exposure (*p* < 0.001).

**Conclusions:**

Both YogaFit and seated rest were effective at acutely reducing state anxiety post-condition, but not at preventing an induced anxiety response post-exposure. Following exposure to the emotionally stimulating pictures, there was a shift from the high frequency-domain to the low frequency-domain and an increase in the time-domain measure of HRV for both the YogaFit and the quiet rest condition.

**Trial registration:**

Retrospectively registered 2/16/2018, clinicaltrials.gov, Identifier: NCT03458702.

**Electronic supplementary material:**

The online version of this article (10.1186/s12906-018-2343-1) contains supplementary material, which is available to authorized users.

## Background

Anxious individuals show elevated cardiovascular responses and slower post-stressor recovery compared to healthy individuals [1]. In this case, the physiological systems of the body are considered less flexible, or desensitized, which is often characterized by a decrease in heart rate variability (HRV) [2, 3].

Anxiety has been effectively manipulated in laboratories using the International Affective Picture System (IAPS) [4]. Experiments using pictures provide a research database regarding affective reactivity and prompt autonomic and somatic reflexes [5]. Anxiety is often psychologically assessed using Spielberger’s State-Trait Anxiety Inventory (STAI) [6]. Physiologically, measures of heart rate (HR) and HRV parallel changes in emotion and anxiety, such that anxiety correlates with decreases in HRV and increases in HR [7].

It has been reported that yoga may improve both the physiological and psychological coping response to stressors [8] by improving HRV and decreasing subjective levels of stress [9–11]. YogaFit is based on a safe and inclusive approach: Some of the traditional yoga poses have been modified to lower the risk of injury and increase the accessibility of yoga for all ages. This type of yoga is often referred to as “flow” yoga because the poses move fluidly with the breath, creating strength, flexibility, endurance, and balance for greater health and mental awareness, thus the term, YogaFit [12].

Smith (2013) reported that an acute session of moderate intensity cycle ergometry, but not a similar session of seated rest, could prevent the rise in anxiety following exposure to IAPS picture stimuli [4]. However, other studies have found that an acute period of rest reduces anxiety as efficiently as an acute exercise session [4, 13–16]. Thus, it may be that the time away from sources of stress has the potential to alter anxiety levels regardless of what activity (e.g. rest, aerobic exercise) is done during that time [13] . Although, the effectiveness of an acute yoga session on anxiety and autonomic balance in response to a laboratory imposed stressor has not been investigated: Nor has the westernized version of yoga, YogaFit [12], been evaluated for its effectiveness in reducing anxiety.

Therefore, the purpose of this study was to examine the effect of an acute bout of YogaFit versus an acute period of seated rest on state anxiety and physiological correlates of anxiety, namely HR and HRV. The study design executed by Smith [4] was emulated, substituting YogaFit for cycle ergometry and using seated rest for the control group. It was hypothesized that an acute bout of YogaFit would reduce state anxiety and increase HRV to a greater degree than a resting condition [9–11]. We also hypothesized that YogaFit would provide a greater short-term protection from the effects of arousing emotional stimuli on state anxiety and stress reactivity (HR) compared to a resting condition [4, 12].

## Methods

### Participants

**Fig. 1:**
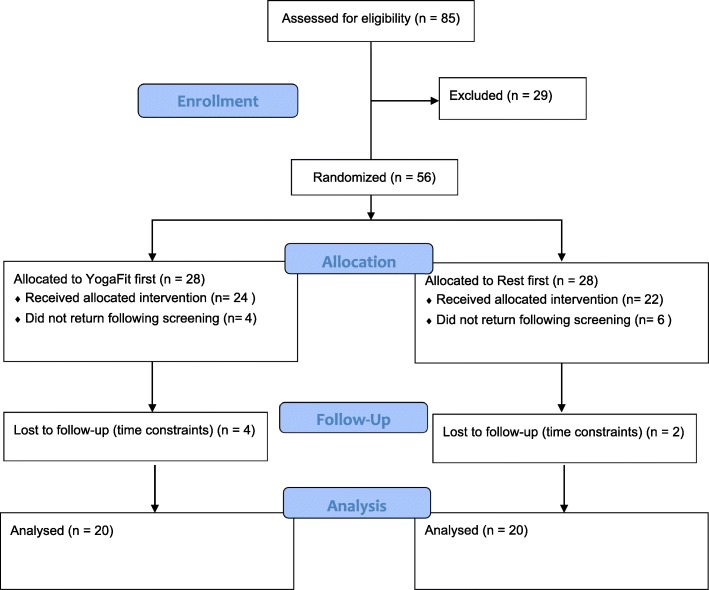
The Consort Flow Diagram

We assessed the eligibility of 85 women recruited from yoga classes at the university. Out of the 85 women who completed the pre-screening process, 29 were excluded from the study. Fifty-six women were eligible and were enrolled in the study. The exclusion criteria included women who: (a) were less than 18 or more than 25 years of age; (b) were suffering from any medical condition that would influence the results or compromise safety during training—such as disorders effecting balance, or pregnancy; (c) were taking antidepressant or anti-anxiety medications; (d) were clinically diagnosed with generalized anxiety disorder in the previous 6 months; (e) were not within the normal range (± 1 *SD* from the *M*) for female college students for trait anxiety according to Spielberger’s Trait Anxiety Inventory [(STAI-Y2); range: 40.40 ± 10.15] [6]; (f) were not within normal (minimal to mild) levels of depression according to the Beck Depression Inventory [(BDI); range: 0–18] [17]; (g) had an abnormal menstrual cycle (cycles occurring less than every 26 to 35 days and lasting less than 2 or more than 7 days); (h) were considered high-risk for dual energy x-ray absorptiometry (DEXA) based on a standardized questionnaire approved by the University Radiation Safety Committee; and (i) were not familiar with yoga or had not participated in at least 3 yoga practice sessions. The purpose for excluding participants above the normal range on the STAI-Y2 and those with above normal to mild levels of depression was to replicate the characteristics of participants in Smith’s study (2013) [18]. Of the 56 eligible participants, 16 women did not complete all required sessions of the study. Forty healthy, female college students completed the study (see Fig. 1).

### Design

The design was a repeated-measures randomized crossover trial. Each study participant completed 4 different sessions on 4 different days: (day 1) participant screening; (day 2 or 3) study condition A (seated, quiet rest); (alternate day 2 or 3) study condition B (moderate-intensity YogaFit); and (day 4) a session to rate IAPS pictures (14) and undergo a single DEXA scan [Prodigy/301776R. G.E. Medical Systems-Lunar DEXA Scanner, Fairfield, CT, USA]. Utilizing a simple randomization process, pparticipants were randomized in a 1:1 ratio to participate in either the YogaFit condition or the quiet rest condition. The study conditions were counterbalanced and alternated using odd and even numbers to begin the sequence. Participants were not informed if the first condition was A or B. Self-reported anxiety levels and 10 min of continuous physiological data were collected: (a) baseline; (b) post-condition; and (c) post-exposure for both condition A and B. All participants completed both the psychological and physiological assessments.

Both experimental conditions A and B were completed in 90 min. To ensure compliance, participants were observed through a glass window every 10 min during study conditions. Testing time and procedures were identical with the exception of the experimental condition on days two and three.

### Procedures

The study was approved by the Texas Tech University Protection of Human Subjects Institutional Review Board (IRB) Committee (Reference Number for the Approved Study: 504055) and was retrospectively registered at clinicaltrials.gov (Identifier No. NCT03458702). All participants signed the approved institutional IRB consent form before pre-screening. This was a 2-way randomized crossover trial, with each participant completing 2 separate study conditions (A or B) on separate days: YogaFit or quiet rest. All participants were familiarized with the videotaped YogaFit session before participating in the study. Participants were instructed to arrive for all visits dressed in loose fitting athletic wear and were asked to refrain from all food, alcohol, caffeine, gum chewing or the use of tobacco products 3 h before their scheduled meeting in the laboratory. During pre-screening, participants completed: (a) a standard health history questionnaire; (b) the STAI-Y2 [6]; (c) the BDI [17] and (d) a custom DEXA safety questionnaire. Qualified participants were then asked to track their menstrual cycle using a menstrual log and e-mail the primary investigator on day 1 of the follicular phase of their menstrual cycle, thus using the first day of the women’s menstrual cycle to generate an unpredictable random sequence and to eliminate any sequence bias. The participant was then scheduled for condition A first or condition B first. The conditions were counterbalanced to control for order effects, with odd numbers beginning condition A first and even numbers beginning condition B first. Previous studies suggest that menstrual cycle has an effect on anxiety levels and autonomic nervous system function [19, 20]. To control for the effects of the menstrual cycle on anxiety, participants completed condition A and condition B within a 7-day period during the follicular phase of their menstrual cycle (specifically days 1–10). Participants were also asked to maintain pre-study physical activity levels during the 7-day period. All testing was completed by day 10 of their menstrual cycle.

State anxiety was psychologically quantified using a reliable and validated assessment of anxiety, Spielberger’s State-Trait Anxiety Inventory (STAI-Y1) [6, 21]. Instruments for testing all physiological variables were calibrated and used by the same investigator in order to control possible inter-tester variation. During both condition A and B, a ProComp Infiniti System with Biograph software version 6.0 was used to measure HR and indices of HRV at a sampling rate of 2048 cycles per sec at the 3 different time points. Three electrodes were placed on the participants to monitor HR as specified by the ProComp Infiniti System. The negative electrode was placed in the right shoulder fossa below the clavicle, the positive electrode was placed medially on the thorax below the sternum, and the ground electrode was placed in the left shoulder fossa below the clavicle. Indices of HRV included the time-domain variable, root mean square of successive differences (RMSSD) and two frequency-domains: the low frequency power bands and the high frequency power bands in normalized units (LFNU and HFNU, respectively). Normalized units in the frequency domain emphasize the controlled and balanced behaviour of the two branches of the autonomic nervous system [22, 23]. Frequency-domains describe the periodic oscillations of the HR signal decomposed at different frequencies and amplitudes in the hearts rhythm. LFNU is a positive oscillation (a sine wave) from 0.04–0.15 Hz and HFNU is a positive oscillation from 0.15–0.40 Hz. Measures of HR and HRV were analysed using CardioPro Infinity-HRV Analysis Software Module-SA7J90 [ProComp Infiniti System, Montreal, Quebec, Canada: Cimetra LLC].

An accelerometer using the ActiLife 6 Software and a Polar Heart Rate Sensor were strapped to the participant and used to monitor the intensity level of both study conditions A and B. The accelerometer was used only to record HR data and no other accelerometer data were analysed. Participants were then informed of the experimental condition. For study condition A (quiet, seated rest), participants sat on a yoga mat in a cross-legged position for 30 min in a quiet laboratory setting. For study condition B [YogaFit Vinyasa Flow (referred to as YogaFit in this manuscript)], participants followed, via digital versatile disc, a standardized YogaFit format choreographed by a certified American Council of Exercise Instructor and Registered Yoga Alliance Teacher. YogaFit was performed in the same laboratory setting and lasted 30 min. There are 7 principles of alignment that are applied in every YogaFit session: (1) establish a base and dynamic tension; (2) create core stability; (3) align the spine; (4) soften and align the knees; (5) relax shoulders back and down; (6) hinge at the hips; and (7) shorten the lever. The YogaFit essence is based on: Breathing, Feeling, Listening to the Body, Letting Go of Competition, Judgement, Expectations, and Staying in the Present Moment. The movements included in each specific phase were chosen from Beth Shaw’s YogaFit Vinyasa Flow Series [12]. Sample movements included in each phase are given, but the list is not inclusive. *Mountain 1*: (5 min) knees to chest; flowing bridge; cat and cow; spinal balance, flowing child’s pose; Valley 1 (5 min): variations of sun salutations; *Mountain 2* (10 min): standing forward fold, monkey, gorilla, chair, sunflower, sun pose, standing straddle splits, pyramid, triangle; side angle pose sequence, side lying plank; *Valley 2* (5 min): standing balances – pigeon, tree, dancer; standing split; warrior 3; and *Mountain 3* (5 min): bound angle pose, big toe wide boat, dead bug, alternate nostril breathing, palm warming, eye rolls, meditation. Breath was an integral part of every movement with specific breath rates for each phase of the session. The objective was to move the body with intention and purpose and be present in the body. To measure intensity of the YogaFit routine, ratings of perceived exertion (RPE) [24] were recorded following both condition A and B. The accelerometer and heart rate sensor were removed after the study condition. After both study conditions, participants again completed STAI-Y1 and 10 min of post-condition data were collected.

Following the protocol for picture viewing used by Smith [4], participants then viewed 90 emotionally arousing pictures from the IAPS after both study conditions A and B. Pictures were viewed on a 70″ × 70″ portable projection screen. The 90 pictures were arranged in 3 blocks of 30; each block contained 10 pictures from each valence category. Valence is a term used in psychology when discussing emotions and refers to the pleasantness (positive valence) or unpleasantness (negative valence) of an object or experience [25]. Among the 90 pictures used, 30 were pleasant (15 erotica and 15 babies, families, and cute animals); 30 were neutral (15 neutral people and 15 neutral objects and scenes); and 30 were unpleasant (15 threat and 15 mutilation) based on normative ratings of valence [5]. No more than two pictures from the same category appeared consecutively. Two different picture orders were constructed and counterbalanced across testing day and experimental condition. Each picture was shown for 4-s, followed by a 12-, 14-, or 16-s inter-picture interval, which consisted of a centrally located fixation cross. The total picture-viewing time, including brief breaks between each picture block, was approximately 30 min. Participants were instructed to look at each picture the entire time it was on the monitor and to subjectively categorize each picture as pleasant, neutral, or unpleasant using a response pad [E-Prime 2.0, Psychology Software Tools, Inc., Pittsburgh, PA, USA] resting on their lap. These ratings were not used to compare to the normative ratings, but rather, the purpose of categorizing the pictures was to ensure that participants concentrated on the pictures. Immediately after viewing the pictures, participants again completed STAI-Y1 and 10 min post-exposure data were collected.

In accordance with the Center for the Study of Emotion and Attention, participants were also asked to rate the IAPS images in order to further image standardization. Participants rated each of the 90 pictures (hard copy, one picture per page in a standard, self-paced order) using the Self-Assessment Manikin (SAM) during visit 4 to the laboratory. Additionally, participants underwent a single DEXA scan for body composition assessment.

### Data analysis

Sample size was based on previous research with similar methods [18]. All analyses were computed using the Statistical Package for Social Sciences (IBM SPSS Statistics Version 21). Means (*M*) and standard deviations (*SD*) were computed for descriptive statistics (age, height, weight, body mass index [BMI], body fat percentage, STAI-Y2, and BDI scores). Dependent t-tests were run on baseline measures to ensure that there were no significant initial differences between conditions. Furthermore, intraclass correlation coefficient (ICC) analysis was run on the baseline STAI-Y1 scores from both conditions to evaluate test/re-test reliability and sensitivity of the STAI-Y1. Pearson product-moment correlation coefficient (*r*) tests were computed between all dependent variables of interest (STAI-Y1, HR and all indices of HRV) to ensure that the variables were distinct variables. A correlation of 0.7 or higher was used to indicate a degree of correlation (49% of variance could be attributed to the other variable) to justify the use of a MANOVA. A split-plot 2 (condition: seated rest or yoga) X 3 (time: baseline, post-condition and post-exposure) repeated measures ANOVA for time was used to compare the effect of an acute YogaFit session and a quiet resting condition on the variables of interest unless there was a correlation of 0.7 or higher. If a correlation of 0.7 or higher existed, a split-plot MANOVA with 2 conditions (seated rest or yoga) X 3 (time: baseline, post-condition and post-exposure) was used to analyse the correlated variables. Appropriate follow up tests were considered to be separate split plot ANOVAS for the dependent variables. Significance for all tests was considered to be at the 0.05 level.

A two-sample *t*-test was used to determine if the two population means for the normative ratings and our participant IAPS picture ratings were significantly different. In calculating the degrees of freedom, it was assumed that the sample variance was equal since both populations were college-aged females. The level of significance for a two-tailed test at the 0.05 level for 88 *df* [*df* = (*n*_1_ + *n*_2_)-2] was 2.021 (Critical Value of t).

### Results

Of the 85 women screened, 56 were eligible. Of these, 28 were randomized to each group (to condition A or B first): 10 did not return following screening, 24 completed the YogaFit condition but of these, 4 did not return for the resting condition and 22 completed the resting condition but of these, 2 did not return for the YogaFit session. Participant characteristics are displayed in Table 1. T-tests indicated no significant difference in participant characteristics collected at screening between participants who completed and did not complete the study (data not shown). Participants were in the normal range for BMI (range: 18.5–24.9 kg/m^2^), trait anxiety (STAI-Y1) and reported levels of depression on the BDI, but in the very poor range for body fat percentage for their age. Dependent t-tests on all baseline measures verified that there were no significant initial differences between the two conditions for STAI-Y1 (*t* = 0.975), HR (*t* = − 0.871), RMSSD (*t* = − 0.147), LFNU (*t* = − 0.101), or HFNU (*t* = 0.100). Thus, no covariates were required for the main analyses. Supporting these findings, ICC analysis on baseline STAI-Y1 scores indicates a good correlation between repeated measures (ICC = .693). Means and SD for all of the dependent variables can be found in Table 2.

**Table 1:** Participant Characteristics

Variable	M	SD
Age (years)	20.18	1.97
Height (cm)	165.83	7.54
Weight (kg)	63.63	9.36
BMI (kg/m^2^)	23.21	3.12
Body fat (%)	34.94	7.58
STAI-Y2 score	39.05	5.91
BDI score	7.68	4.21

*BMI* body mass index, *STAI-Y2* Trait Anxiety Inventory, *BDI* Beck Depression Inventory

**Table 2:** Means and standard deviations of examined variables (*N* = 40)

Variable	Yoga		Rest	
	*M*	*SD*	*M*	*SD*
Baseline STAI-Y1, Ŧ	33.48	9.41	33.53	10.63
Post-condition STAI-Y1	27.43	7.05	29.78	7.82
Post-exposure STAI-Y1	33.18	10.75	34.48	10.34
Baseline HR, *, Ŧ	72.48	10.61	73.59	11.25
Condition HR	101.01	22.65	69.35	10.61
Post-condition HR	74.51	10.39	70.43	9.77
Post-exposure HR	71.69	9.73	70.17	9.65
Baseline RMSSD^a^, Ŧ	61.32	30.80	61.87	33.98
Post-condition RMSSD	62.41	36.33	75.66	40.67
Post-exposure RMSSD	72.75	43.11	79.41	42.49
Baseline LFNU, Ŧ	53.50	17.13	53.75	17.93
Post-condition LFNU	52.56	16.07	54.50	17.77
Post exposure LFNU	58.42	13.86	56.25	15.18
Baseline HFNU, Ŧ	46.49	17.13	46.25	17.93
Post-condition HFNU	47.19	16.22	44.55	17.89
Post-exposure HFNU	41.58	13.86	43.55	15.15
Post-condition RPE	10.83	1.66	6.05	0.22

*STAI-Y1* State Anxiety Inventory, *HR* heart rate, *RMSSD* root mean square of successive differences in RR intervals, *LFNU* low-frequency power, *HFNU* high-frequency power, *RPE* Ratings of Perceived Exertion; ^a^ = significant condition x time interaction; Ŧ = significant interaction for time, *P* < 0.05

Statistical analyses were conducted including data from the 6 non-completers. Data for the missing variables was imputed using a multiple regression analysis, the Poisson Pseudo Maximum Likelihood (PPML) method, y = mx + c [26]. State and Trait Anxiety from the screening session were used to predict the subjective (STAI-Y1) and the physiological (HRV) responses to both rest and YogaFit conditions [7]. Height, weight and age were used to predict HR and RPE [27]. The statistical models described above were then re-run using the predicted variables. Means and SD for all of the dependent variables with imputed data are found in Additional file 1: Table S1.

Means and SD for normative and participant ratings of IAPS pictures using the SAM can be found in Table 3. There was no statistical difference between the normative and participant ratings for each picture category.

**Table 3:** Affective ratings of picture stimuli from IAPS

	Normative Ratings *N* = 50		Participant Ratings *N* = 40		Test Statistics
	*M*	*SD*	*M*	*SD*	
Pleasant Picture Valance Rating	7.80	0.61	6.88	1.80	0.1523
Pleasant Picture Arousal Rating	5.90	0.55	2.99	2.32	0.3863
Pleasant Picture Dominance Rating	5.77	0.93	3.93	2.29	0.2330
Neutral Picture Valance Rating	5.01	0.31	5.36	1.10	−0.0967
Neutral Picture Arousal Rating	3.48	0.98	1.28	0.85	0.5047
Neutral Picture Dominance Rating	5.50	0.94	2.94	2.0	0.3609
Unpleasant Picture Valance Rating	1.80	0.40	1.76	1.25	0.0096
Unpleasant Picture Arousal Rating	6.77	0.59	3.17	2.97	0.3770
Unpleasant Picture Dominance Rating	3.41	1.02	6.56	2.57	−0.3568

A two-sample t-test determined no significant difference between mean self-assessment manikin (SAM) ratings to pleasant, neutral and unpleasant pictures between study participants and the published normative values for college women. The critical value needed to reach significance was 2.021

### State anxiety (STAI-Y1)

**Fig. 2:**
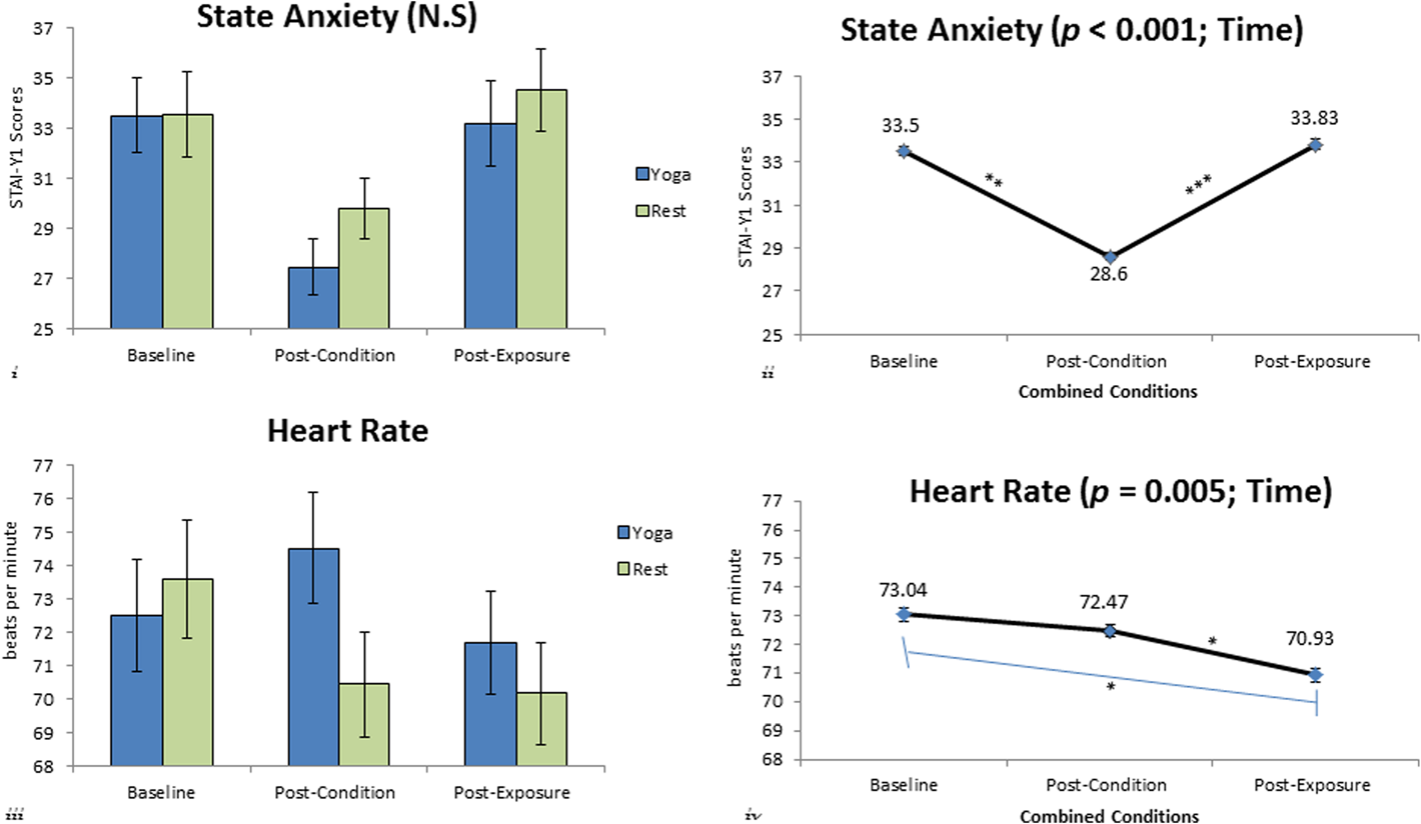
(i) State anxiety scores at baseline, post-condition, and post-exposure for the yoga and rest conditions. No significant difference between the yoga and rest conditions. (ii) Combined state anxiety scores at baseline, post-condition and post-exposure. Significant decrease, **p = 0.001, between baseline and post-condition and significant increase, ***p < 0 .001, between post-condition and post-exposure. (iii) Heart rate (HR) at baseline, post-condition and post-exposure for the yoga and rest conditions. Significant interaction between condition & time, ***p < 0 .001. (iv) Combined HR for both conditions at baseline, post-condition and post-exposure. Significant decrease between post-condition and post-exposure, ^*^p = 0 .020 and between baseline and post-exposure, * p = 0 .033. Note symbols or abbreviations on graphs: N.S.- not significant, *p < 0.05, **p = 0 .001, and ***p < 0 .001. Error bars = SEM

The 2 X 3 split-plot repeated measures ANOVA, F(2, 78) = 14.836, *p* < 0.001, η2 = .276, SP = .997, had a significant main effect for time. There was not a significant condition x time interaction, nor was there a significant main effect for condition. The results for the Bonferroni post hoc comparisons indicated that the mean state anxiety scores were significantly decreased, *p* = 0.001, 95% CI [2.71, 7.09], between the time points baseline (33.50 ± 1.11) and post-condition (28.60 ± 0.84) and returned to baseline levels, *p* < 0.001, [− 8.067 to − 2.383], between the time points post-condition and post-exposure (33.83 ± 1.17), as indicated in Fig. 2.

### Heart rate (HR)

The 2 X 3 repeated measures ANOVA for HR had a significant condition x time interaction, F(2, 78) = 10.655, *p* < 0.001, η2 = .215, SP = .987. As expected, HR post-condition for the YogaFit condition was significantly higher compared to HR post-condition for rest. There was also a significant main effect for time, F(2, 78) = 5.656, *p* = 0.005, η2 =. 127, SP = .849. Results for the Bonferroni post hoc comparisons indicated that heart rate was significantly decreased, *p* = 0.033, [0.132, 4.092], between baseline (73.04 ± 1.22 bpm) and post-exposure (70.93 ± 1.08 bpm), *p* = 0.020, 95% CI [0.198, 2.890], and between post-condition (72.45 ± 1.14 bpm) and post-exposure (70.93 ± 1.08 bpm). See Fig. 2.

### Heart rate variability (HRV)

The 2 X 3 repeated measures MANOVA was statistically significant for a condition x time interaction, *p* = 0.028, η2 = .088, SP = .813. There was also a significant main effect for time, *p* < 0.001, η2 = .248, SP = 1.00. However, the 2 X 3 repeated measures MANOVA did not have a significant main effect for condition.

#### Root mean square of successive differences (RMSSD)

The condition x time interaction for RMSSD, F(2,78) = 3.314, *p* = 0.042, η2 = .078, SP = .612, was significant. There was also a significant main effect for time, F(2, 78) = 15.877, *p* < 0.001, η2 = .289, SP = .999. Results for the Bonferroni post hoc comparisons indicated that RMSSD was significantly increased between baseline (61.59 ± 4.77 ms) and post-condition (69.03 ± 5.59 ms), post-condition and post-exposure (76.08 ± 6.29 ms) and between baseline and post-exposure, *p* = 0 .019, 95% CI [− 13.877, − 1.004], *p* = 0 .007, [− 12.466, − 1.621] and *p* < 0.001, [− 21.780, − 7.187], respectively . There was only a significant main effect for condition when the non-completers’ variables and imputed data were accounted for in the analysis, F(1,45) = 6.32, *p* = 0.016, η2 = .123, SP = .692 (Additional file 1: Table S1).

#### Frequency domain variables

**Fig. 3:**
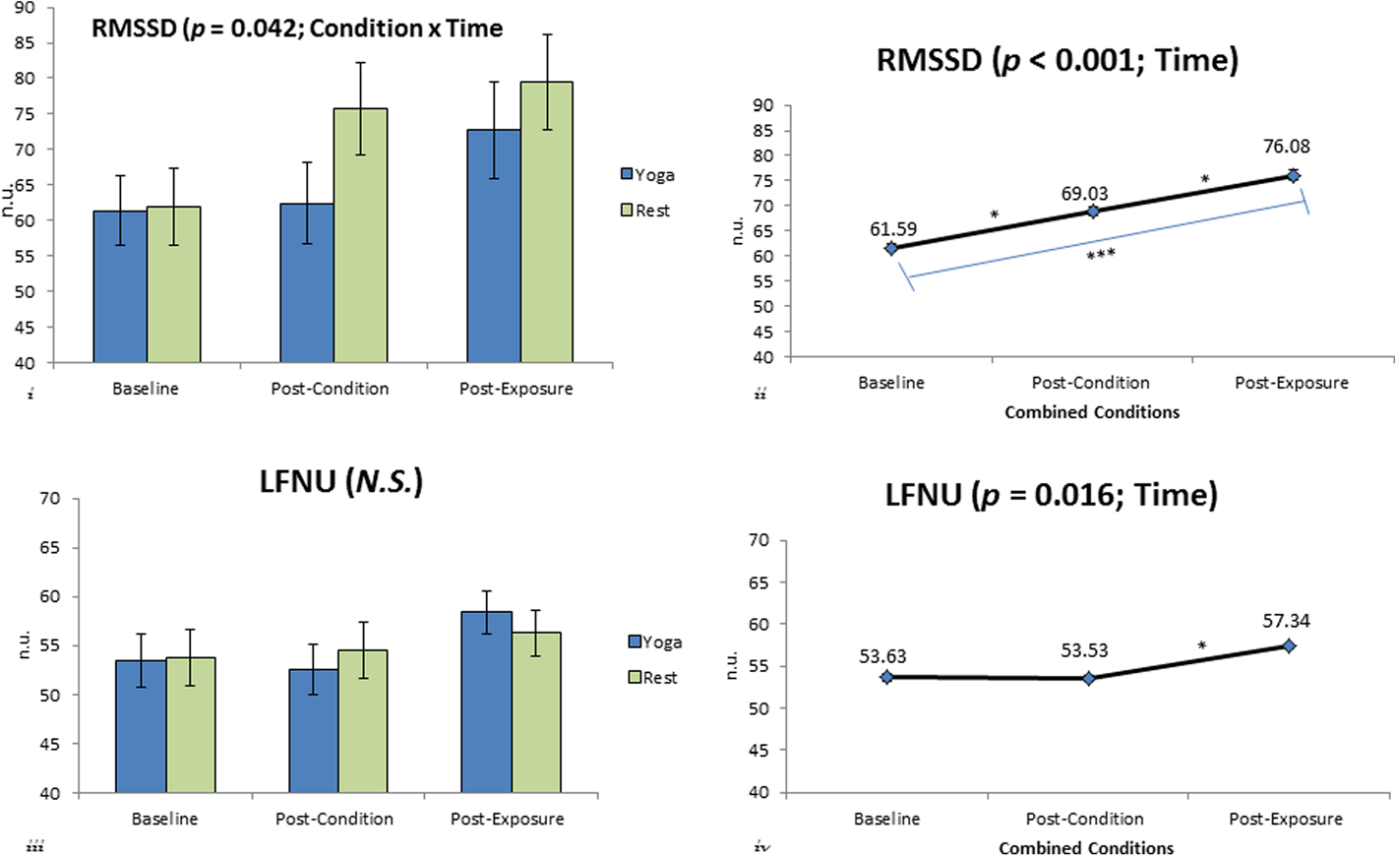
(i) Root Mean Square of Successive Differences in RR Intervals (RMSSD) at baseline, post-condition and post-exposure for the yoga and rest conditions. Significant condition x time interaction, * p = 0 .042. (ii) Combined RMSSD at baseline, post-condition and post-exposure. Significant increase between baseline and post-condition *(p = 0 .019*), post-condition and post-exposure (*p = 0 .007), and between baseline and post-exposure,*** p < 0 .001. (iii) Low-frequency power in normalized units (LFN) at baseline, post-condition and post-exposure for the yoga and rest conditions. No significant difference between the yoga and rest conditions. (iv) Combined LFN at baseline, post-condition and post-exposure. Significant increase,* p = 0 .008, between post-condition and post-exposure. *Note symbols or abbreviations on graphs:* N.S.- not significant, *p < 0 .05, **p = 0 .001, and ***p < 0 .001. Error bars = SEM

There was neither a significant condition x time interaction, nor a main effect for condition for LFNU or HFNU. However, there was a significant main effect for time, for both LFNU, F(2,78) = 4.393, *p* = 0.016, η2 = .101, SP = .743 and HFNU, F(2, 78) = 3.823, *p* = 0.026, η2 = .089, SP = .679. Results for the Bonferroni post hoc comparisons indicated that LFNU was significantly increased, *p* = 0.008, 95% CI [− 6.760, −.850], between post-condition (53.53 ± 1.89 n.u.) and post-exposure (57.34 ± 1.62 n.u.), see Fig. 3. Results for the Bonferroni post hoc comparisons indicated that HFNU was significantly decreased, *p* = .025, 95% CI [.335, 6.275] between post-condition (45.87 ± 2.32) and post-exposure (42.57 ± 1.99). HFNU is not displayed in a figure since it is the reciprocal of LFNU.

## Discussion

The purpose of this study was to analyse the effectiveness of an acute session of YogaFit on state anxiety and physiological correlates that reflect stress reactivity following exposure to emotional stimuli. We hypothesized that the YogaFit session would provide greater protection to an emotional response evoked by visual stimuli than seated rest. The results of this study did not support this hypothesis. When examined for the three different time points, STAI-Y1 scores were significantly decreased between baseline and post-condition and returned to baseline post-exposure. Post-condition decreases in STAI-Y1 scores indicate that both study conditions were effective at reducing anxiety levels acutely, supporting the hypothesis that time away from sources of stress (i.e. the time out hypothesis) is an important factor in reducing anxiety [13]. However, the decrease in anxiety did not persist 30–40 min post-condition, as expected, so our hypothesis that YogaFit would provide protection to emotionally arousing stimuli and that this protection would be greater than seated rest post-exposure was not supported. Perhaps it could be said that both seated rest and YogaFit provided a marginal protective effect to the emotionally arousing stimuli, since there was not a significant difference between baseline and post-exposure STAI-Y1 scores. Of note, baseline STAI-Y1 scores were similar between the two groups (ICC = .639), indicating similar starting points for both conditions as well as reliability of the STAI-Y1. In addition, there were other physiological correlates normally associated with reduced anxiety, such as a decrease in HR, and an increase in RMSSD post-exposure in both groups. We also saw an increase in the low frequency-domain following the exposure to emotionally arousing stimuli.

While other studies did not induce a stressor during or following an exercise bout, they did find that anxiolytic effects are sustained for a longer period following aerobic exercise compared to their control condition [16], and that effects are maintained longer at higher intensity aerobic exercise, 80% VO_2_ Max compared to 60% VO_2_ Max [15]. The average reported RPE for the YogaFit condition was 10.83 ± 1.66 and is reflective of a *fairly light* level of exertion [24]. Accordingly, HR recorded during YogaFit (101.01 ± 22.65 bpm; see Table 2) is roughly 50% of maximum HR based on participant age and would be considered *light intensity* [28]. Therefore, it may be that the intensity of the YogaFit session was not sufficient to produce reductions in post-exposure STAI-Y1 scores [29].

Increased anxiety is paralleled with increases in HR [7, 30] and decreases in HRV [30, 31]. In our study, we report an increase in RMSSD following exposure to emotionally provoking stimuli (see Fig. 3). RMSSD is thought to represent parasympathetic stimulation [32, 33] and reflect alterations in autonomic tone that are predominantly mediated by the vagus nerve [32]. Thus, increases in RMSSD parallel the post-exposure decrease in HR [22] reported in our study. Furthermore, post-exposure increases in RMSSD were greater for rest, further supporting the time-out hypothesis.

Our findings are somewhat unique in that we saw an increase in the low frequency-domain (0.04–0.15 Hz) and a decrease in the high frequency-domain (0.15–0.40 Hz). Interpretation of the low frequency component of HRV is controversial [34] but is believed to reflect both sympathetic and parasympathetic activity, specifically it may provide insight into the link between cardiac sympathetic nerve activity and baroreflex sensitivity [34]. The low frequency-domain is also considered to be when the interplay of the body’s natural rhythms are most effective and HRV is increased [2, 3]. In our study, we saw concurrent increases in RMSSD and LFNU power in both the yoga and rest conditions.

Indeed, the goal of resonance breathing training or HRV training is to induce a peak in the HR amplitude at 0.08 to 0.1 Hz, which is in the low frequency-domain, also known as the meditators’ peak. In training, the development of a stable, sine-wave-like pattern at approximately 0.1 Hz is the key marker of the psychophysiological mode where HRV is maximized [35, 36]. Thus, engaging in rest or any activity (i.e. yoga) that increases the power in the low frequency-domain during training would concurrently maximize HRV.

When preceded by 30 min of YogaFit or seated quiet rest, oscillations in the body shifted to maintain homeostasis or to counteract the physiological response to viewing the emotionally arousing pictures. Reciprocally, this resulted in a decrease in HR, an increase in low frequency power, and an increase in HRV. This shift in frequency in our participants is of significance since they had high percentages of body fat. It has been suggested that high body fat percentage may be associated with decreased low frequency power [37].

### Limitations

There are limitations that may have affected study outcomes. First, participants used a response pad to rate each picture during emotional exposure [4]. The motor control required for the task may have diverted attention from the stimulus and had an effect on emotional response [38]. Second, post-exposure scores (combined 34.48 ± 10.34) in our study were seemingly lower than the STAI-X1 (X and Y are strongly correlated) scores reported by Spielberger for college-age females during an exam (43.69 ± 11.59) or after viewing a disturbing movie (60.94 ± 11.99) [6]. This could indicate that: (1) the stressor was not of sufficient magnitude to induce STAI-Y1 scores significantly greater than those seen at baseline; or that (2) the exclusion criteria for the STAI-Y2 scores excluded individuals who would have had a greater arousal rating. Third, STAI-Y1 scores and physiological stress reductions are expected to last for 2–4 h after cessation of activity [39]. Changes in HRV may have persisted or again changed within the 2–4 h period, which was not measured. Furthermore, we did not measure blood pressure and can only speculate that the increase in LFNU, RMSSD, and a concomitant decrease in HR, reflect the oscillation of the baroreceptor closed-loop control system. Fourth, motivation and interest for physical activity, especially yoga, were not evaluated. It is not completely clear in which way the level of motivation towards or interest in yoga may have affected the benefits of yoga and corresponding physiological measures and STAI-Y1 scores. It is known that motivational factors are involved in the stress coping mechanism of exercise [40]. It has been considered that characteristics such as sex, body fat percentage and an individual’s perception of control over a situation may influence the effectiveness of physical fitness on cardiovascular reactivity [41]. Therefore, in addition to motivation, these characteristics could have also influenced the anxiolytic effectiveness of yoga. Fifth, both psychological and physiological responses are difficult to gauge in the absence of control session where participants did not view the IAPS pictures. However, our goal was to replicate Smith’s [4] study as closely as possible. In light of our present findings, we suggest that future studies employ a repeated measures design in which measurements are taken following a time period identical to the viewing of the pictures but that participants do not view the emotionally arousing stimuli. Lastly, results may not be generalizable beyond the specific college-aged population from which the sample was drawn. Furthermore, results may not be generalizable to the male population.

## Future studies

Discrepancies in the literature could be due to differences in resting conditions. For example, Smith [4] had participants rest by sitting on a bike seat as a control condition. In order to accurately determine the anxiolytic effectiveness of rest, investigators need to employ a consistent resting condition. Furthermore, employing a few different conditions that reflect the different aspects of yoga (i.e. the physical, mental and spiritual) may help to elucidate the mechanisms by which yoga stimulates a healthy adaptive response to stressors.

## Conclusion

State anxiety scores indicate that both YogaFit and seated rest were effective at acutely reducing state anxiety post-condition, but not at preventing induced anxiety responses post-exposure. Following exposure to the emotionally stimulating pictures, there was a shift from the high frequency-domain to the low frequency-domain and an increase in the time-domain measure of HRV for both the YogaFit and the quiet rest condition. HR decreased, RMSSD increased, and LFNU increased post-exposure suggesting that the oscillatory components in response to increased anxiety post-exposure shifted to maintain homeostasis.

## Additional file

Additional file 1: **Table S1.** Means and standard deviations of examined variables with imputed data (*N* = 46). Data for the missing variables was imputed using a multiple regression analysis, the Poisson Pseudo Maximum Likelihood (PPML) method, y = mx + c . The statistical models used for the study were then re-run using the predicted variables. Means and SD for all of the dependent variables with imputed data are found in Additional file 1: Table S1. (DOCX 26 kb)

## Abbreviations

BDI: Beck Depression Inventory
BMI: Body mass index
DEXA: Dual energy x-ray absorptiometry
HFNU: High frequency power band
HR: Heart rate
HRV: Heart rate variability
IAPS: International Affective Picture System
LFNU: Low frequency power band
M: Mean
RMSSD: Root mean square of successive differences in R-R intervals
RPE: Ratings of perceived exertion
SAM: Self-Assessment Manikin
SD: Standard deviation
STAI: State-Trait Anxiety Inventory
