# Supplementary material for: The effects of yoga and quiet rest on subjective levels of anxiety and physiological correlates: a 2-way crossover randomized trial
Source: BMC Complement Altern Med. 2018 Oct 17;18:280. doi: 10.1186/s12906-018-2343-1 (PMC6191923; doi:10.1186/s12906-018-2343-1)
Supplement: Supplementary file 1 — Table S1. Means and standard deviations of examined variables with imputed data (N = 46). Data for the missing variables was imputed using a multiple regression analysis, the Poisson Pseudo Maximum Likelihood (PPML) method, y = mx + c . The statistical models used for the study were then re-run using the predicted variables. Means and SD for all of the dependent variables with imputed data are found in Additional file 1: Table S1. (DOCX 26 kb) [file 12906_2018_2343_MOESM1_ESM.docx]

Supplemental file 1: Table S1

*Means and standard deviations of examined variables* *with imputed data* (*N*=46)

| Variable |  | | |  | |  | | |
| --- | --- | --- | --- | --- | --- | --- | --- | --- |
|  | Yoga | | |  | | Rest | | |
|  | *M* | | *SD* |  | | *M* | | *SD* |
| Baseline STAI-Y1, Ŧ | 33.83 | | 9.20 |  | | 33.30 | | 9.92 |
| Post-condition STAI-Y1 | 27.40 | 6.55 | | |  | | 29.72 | 7.31 |
| Post-exposure STAI-Y1 | 33.03 | | 10.04 |  | | 34.41 | | 9.65 |
| Baseline HR, *, Ŧ, £ | 72.66 | | 9.97 |  | | 73.35 | | 10.56 |
| Condition HR | 101.27 | | 19.61 |  | | 69.19 | | 9.31 |
| Post-condition HR | 74.69 | | 9.79 |  | | 70.14 | | 9.19 |
| Post-exposure HR | 71.74 | | 9.17 |  | | 69.84 | | 9.10 |
| Baseline RMSSD*, Ŧ, £ | 60.93 | | 29.45 |  | | 63.20 | | 32.01 |
| Post-condition RMSSD | 61.95 | | 34.39 |  | | 76.82 | | 38.14 |
| Post-exposure RMSSD | 72.46 | | 41.56 |  | | 81.02 | | 40.06 |
| Baseline LFNU, Ŧ | 53.62 | | 16.20 |  | | 53.24 | | 16.87 |
| Post-condition LFNU | 52.57 | | 15.39 |  | | 54.06 | | 16.66 |
| Post exposure LFNU | 58.41 | | 13.18 |  | | 55.78 | | 14.31 |
| Baseline HFNU, Ŧ | 46.33 | | 16.19 |  | | 46.76 | | 16.87 |
| Post-condition HFNU | 47.09 | | 15.51 |  | | 45.07 | | 16.80 |
| Post-exposure HFNU | 41.58 | | 13.18 |  | | 44.04 | | 14.29 |
| Post-condition RPE | 10.83 | | 1.56 |  | | 6.05 | | 0.21 |

1STAI-Y1 = State Anxiety Inventory; HR = heart rate; RMSSD = root mean square of successive differences in RR intervals; LFNU= low-frequency power; HFNU = high-frequency power; RPE = Ratings of Perceived Exertion; * = significant condition x time interaction; Ŧ = significant interaction for time, £ = significant interaction for condition, *P* < 0.05.
